# Electronic Health Records Should Support Clinical Research

**DOI:** 10.2196/jmir.7.1.e4

**Published:** 2005-03-14

**Authors:** John Powell, Iain Buchan

**Affiliations:** ^2^Northwest Institute for Bio-Health InformaticsMedical SchoolUniversity of ManchesterManchester M13 9PTUnited Kingdom; ^1^Division of Health in the CommunityWarwick Medical SchoolUniversity of WarwickCoventry CV4 7ALUnited Kingdom

**Keywords:** Medical records, medical records systems, computerized, health services research, epidemiologic research design

## Abstract

One aspect of electronic care records which has received little attention is the potential benefit to clinical research. Electronic records could facilitate new interfaces between care and research environments, leading to great improvements in the scope and efficiency of research. Benefits range from systematically generating hypotheses for research to undertaking entire studies based only on electronic record data. Researchers and research managers must engage with electronic record initiatives to realize these benefits. Clinicians and patients must have confidence in the consent, confidentiality and security arrangements for the uses of secondary data. Provided that such initiatives establish adequate information governance arrangements, within a clear ethical framework, innovative clinical research should flourish. Major benefits to patient care could ensue given sufficient development of the care-research interface via electronic records.

 In the United Kingdom, the government has invested £6200 millionin establishing a National Programme for Information Technology (NPfIT) in the National Health Service (NHS), and further vast resources will be spent on its implementation [1]. This program promises to deliver electronic records, electronic prescribing and electronic booking of appointments underpinned by a modern NHS Information Technology (IT) infrastructure [2]. Of these initiatives, the one with the greatest potential to revolutionize patient care and the working practices of health professionals is the electronic record. This issue of the *Journal of Medical Internet Research* carries a “Viewpoint” article by Gunter and Terry which summarises the benefits of the Electronic Health Record (EHR) [3]. These include the following: medical-error reduction and time saving due to the e-record's availability and legibility; information sharing with patients; and support for clinical decision making. Drawing on the experience of Australia and the United States, Gunter and Terry provide a thorough overview of recent developments in the EHR, and a rigorous examination of the drivers of these developments and the challenges faced by providers.

One aspect of the EHR that is not addressed by Gunter and Terry, and which has received little attention elsewhere, is the great potential of electronic records to benefit clinical research. Research, service-development and public health uses of care records have been referred to as “secondary uses”. In the United Kingdom, the NPfIT is preparing a Secondary Uses Service (SUS) that will become part of the new NHS Information Centre [4]. The confidentiality and security of patient records is an essential consideration [5], especially in the SUS context, where anonymization and pseudonymization of records is planned. Understandably both patients and professionals have raised concerns about the security of electronic records; and it is important that adequate information governance arrangements are established to ensure that confidentiality is protected. The accuracy of records and the quality of data coding must also be assured [6]. Given adequate safeguards, electronic care records could facilitate new interfaces between care and research environments, leading to great improvements in the scope and efficiency of clinical research.

Possible research benefits range from systematically generating hypotheses for research to undertaking entire studies based only on electronic record data. Information for planning studies, such as prevalence and variance of conditions in local contexts could be collected with ease. The patient-owned section of the record could be used by individuals to indicate their general willingness or otherwise to participate in research, or by investigators to alert potential research participants to the existence of a trial. Electronic prompts could signal an attending clinician of a patient's eligibility for an ongoing trial. Simple links from the care electronic record to the trial website could be used to provide further information on the trial for both clinician and patient. Informed consent procedures could be handled systematically under full clinical information and research governances.

National registers of diseases and treatments could be established easily and economically, and with a coherent approach to security across agencies. Epidemiological research could be accelerated and expanded in scope via registers covering well-characterised populations. This would reduce the cost of setting up such studies and provide more timely data that could lead to findings that have greater external validity than the equivalent based on less contemporary data collected in the conventional way. In addition, electronic records which “follow” the patient are likely to provide an efficient method of capturing outcome data in clinical trials and longitudinal studies. This is not an exhaustive list, but it illustrates the enormous potential of electronic records to support clinical research. In the United Kingdom the NPfIT represents an opportunity to develop clinical research that should not be missed.

Researchers and research managers must engage with EHR initiatives to realize such benefits. Programs such as the NPfIT must ensure that clinicians and patients have confidence in the consent, confidentiality and security arrangements for the uses of secondary data. Trust is vital to the practitioner-patient relationship and should not be eroded. Debates around the ”opt-in” or ”opt-out” consent to the use of electronic record data must consider the issue of secondary data usage and clinical research as a population health need. Clinicians and patients must be reassured that no personally identifiable information will be used for research without the consent of the individual. Provided that such programs establish adequate information governance arrangements, within a clear locally-owned ethical framework, such concerns should be addressed and innovative clinical research should be able to flourish. Major benefits to patient care could ensue.

## Abbreviations

EHR: Electronic Health Record
IT: Information Technology
NHS: National Health Service (UK)
NPfIT: National Programme for Information Technology
SUS: Secondary Uses Service
